# Linking Pulse‐Duration‐Controlled Laser Nanostructuring to Oxygen Evolution Kinetics in Fe‐enriched NiOx Electrodes

**DOI:** 10.1002/smll.73897

**Published:** 2026-05-20

**Authors:** Sandra Susan Koshy, Jyotisman Rath, Amirkianoosh Kiani

**Affiliations:** ^1^ Silicon Hall: Micro/Nano Manufacturing Facility Ontario Tech University Oshawa Ontario Canada; ^2^ Department of Mechanical and Manufacturing Engineering Ontario Tech University Oshawa Ontario Canada; ^3^ Department of Chemical Engineering Institute of Chemical Technology – IndianOil Odisha Campus Bhubaneswar Odisha India; ^4^ Davidson School of Chemical Engineering Purdue University West Lafayette Indiana USA

**Keywords:** electrode fabrication, Fe doping, oxygen evolution reaction, transition metal oxide, water splitting

## Abstract

The oxygen evolution reaction (OER) remains the main kinetic and energetic bottleneck in alkaline water electrolysis, motivating scalable and durable electrocatalysts based on earth‐abundant materials. Nickel oxide systems, especially when transformed into NiOOH/NiFeOOH phases, are among the most promising non‐precious OER catalysts; however, conventional synthesis and binder‐based fabrication often restrict control over morphology, active‐site accessibility, and stability. Here, pulse‐duration‐controlled ultra‐short pulsed laser processing (ULPING) is established as a binder‐free and scalable route to directly fabricate nanostructured NiOx electrodes while systematically linking fabrication physics to OER kinetics. By varying pulse duration from 150 ps to 5 ns under otherwise identical irradiation, pulse duration is shown to govern ablation depth, nanostructure growth height, and hierarchical porosity. Shorter pulses produce rough, defect‐rich, broccoli‐like NiOx architectures with high nano‐area gain, whereas longer pulses lead to deeper craters and smoother, melt‐dominated morphologies. Modeling of transient temperature fields and ablation profiles explains the observed topographical evolution. Electrochemical measurements reveal a strong correlation between pulse‐duration‐controlled morphology, redox‐accessible Ni^2^^+^/Ni^3^^+^ active‐site density, and OER performance. The low‐pulse‐duration electrode shows lower overpotential, reduced charge‐transfer resistance, favorable Tafel slopes, and further enhancement after Fe incorporation. Stable operation at 50 mA cm^−^^2^ for 25 h confirms excellent durability and preserved nanostructural integrity.

## Graphical abstract

Pulse‐duration‐controlled ultra‐short laser processing is shown to directly create binder‐free Fe‐enriched NiOx electrodes with tunable porosity, active‐site density, and oxygen‐evolution kinetics. Shorter pulses generate defect‐rich nanostructures that accelerate charge transfer, promote highly active NiFeOOH formation, and deliver durable alkaline water oxidation, revealing fabrication physics as a key lever for catalyst design.

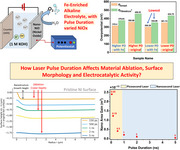

## 1 Introduction

Green hydrogen produced from renewable electricity is increasingly positioned as a central *energy carrier* for deep decarbonization because it can (i) store intermittent wind/solar power in chemical form, (ii) enable long‐duration/seasonal storage, and (iii) provide a low‐carbon reductant and feedstock for hard‐to‐abate sectors (e.g., ammonia/fertilizers, refining, iron/steel, and high‐temperature process heat) where direct electrification is constrained by infrastructure, process chemistry, or operating temperature windows [1, 2]. Among available production pathways, water electrolysis is uniquely compatible with a fully renewable value chain, but its overall efficiency and cost are controlled by electrocatalysis at the two half‐reactions: the hydrogen evolution reaction (HER) at the cathode and the oxygen evolution reaction (OER) at the anode. In alkaline and anion‐exchange–based electrolyzers—often favored for their materials flexibility and potential to avoid precious metals—OER is widely recognized as the kinetic and energetic bottleneck because it involves multi‐electron/proton‐coupled elementary steps and O─O bond formation, leading to substantial activation losses and a large overpotential requirement relative to the thermodynamic 1.23 V (vs. RHE). Consequently, the rational design of OER electrocatalysts remains pivotal for improving electrolyzer voltage efficiency, reducing stack operating costs, and enabling high current density operation under practical conditions [3]. In alkaline media, transition‐metal (oxy)hydroxides and oxides, particularly those based on Ni, Fe, and Co, have emerged as leading earth‐abundant catalyst families because they can access high‐valent metal–oxygen states under anodic polarization while maintaining reasonable corrosion resistance [4].

Among them, NiO/NiOx precursors that transform *in operando* to Ni(oxy)hydroxide (often described as NiOOH‐like phases) are especially compelling owing to the Ni‐centered redox chemistry (Ni^2^^+^/Ni^3^^+^) that couples strongly to oxygenated intermediates, enabling high OER turnover in alkaline electrolyte, and multiple benchmarking studies have shown that optimized Ni–Fe (oxy)hydroxide catalysts can achieve specific activities (normalized to electrochemically active surface area) that are competitive with, and in some cases exceed, noble‐metal oxide references (IrOx/RuOx) under alkaline conditions, while offering a clear advantage in elemental abundance and cost relative to Ir and Ru [3]. A canonical demonstration of the performance ceiling of Ni–Fe systems is the NiFe layered double hydroxide (NiFe‐LDH) motif, where Fe incorporation into Ni hydroxide yields exceptionally active and durable OER electrocatalysts [5].

Despite these advances, translating intrinsic NiOx/Ni(Fe)OOH activity into manufacturable electrodes faces persistent challenges: many high‐performing NiOx morphologies are produced via solution/chemical synthesis routes (hydrothermal/solvothermal growth, precipitation, template or surfactant methods) that require multiple precursors, pH/complexant control, and post‐treatments, and the resulting powders are frequently integrated into electrodes using inks that rely on polymeric binders and dispersants (e.g., Nafion, PTFE, PVDF). Such binders can dilute catalyst utilization by blocking active sites, introduce additional interfacial resistances, and complicate gas disengagement and wetting. Such issues become increasingly consequential at industrially relevant current densities where bubble dynamics and transport losses are nontrivial [3]. These limitations have motivated intensified interest in *binderless* catalyst–electrode architectures and manufacturing routes that directly create robust catalyst layers on conductive substrates, including electrodeposition, ALD/CVD/PVD, and laser‐based structuring/oxidation [6, 7, 8].

However, many deposition‐based methods require stringent precursor delivery, vacuum infrastructure (for ALD/PVD/CVD), tight process windows, and/or sophisticated thickness/stoichiometry control to ensure reproducibility across areas beyond laboratory scale; moreover, achieving simultaneous control over (i) catalyst mass loading, (ii) hierarchical porosity, and (iii) spatial arrangement of nanostructures—while maintaining strong adhesion and low contact resistance—remains nontrivial for large‐area electrodes. In this context, pulsed laser ablation/laser nanostructuring provides a physically direct, chemical‐lean pathway to fabricate binder‐free metal/metal‐oxide electrode assemblies. By delivering high peak powers in short temporal bursts, laser processing can generate high‐surface‐area textures and promote near‐surface oxidation and defect formation without requiring catalyst inks, binders, or multi‐reagent synthesis, while offering straightforward patterning and scalability by beam scanning over centimeter‐scale areas [8, 9, 10, 11, 12]. Importantly, the *pulse duration* (the intrinsic temporal width of each pulse) governs the balance between non‐equilibrium energy deposition and thermal diffusion. Femtosecond–picosecond regimes can localize energy within electron–phonon relaxation timescales and minimize the heat‐affected zone (“cold” ablation), whereas nanosecond pulses more readily couple to melting/re‐solidification and thermochemical oxidation, thereby altering ablation efficiency, recast formation, oxide phase evolution, and ultimately the resulting surface morphology [12, 13, 14].

Foundational work comparing femtosecond/picosecond/nanosecond ablation established that pulse duration strongly modulates ablation mechanisms and material removal characteristics, and modern simulation frameworks, often based on two‐temperature models, further rationalize how ultrashort pulses drive distinct micro/nanostructure formation and periodic surface textures [13]. Recent electrolysis‐relevant demonstrations underscore that laser texturing of Ni‐based substrates can tune OER performance through controlled phase/structure evolution (e.g., nanosecond laser texturing of Ni for OER [15], and that femtosecond‐laser‐induced structuring of porous nickel architectures can create catalyst‐layer‐substituting anodic surfaces for alkaline OER, consistent with broader observations that laser‐induced periodic surface structuring can reduce cell voltages via field localization and enhanced interfacial kinetics [9, 16].

Parallel to fabrication questions, NiOx OER science faces a second, intertwined complexity: the outsized role of Fe, often present at ultra‐trace levels in commercial KOH, in transforming nominal “NiOOH” into mixed NiFe oxyhydroxide motifs that can dominate OER kinetics. Classic electrochemical evidence showed that even ppm‐to‐sub‐ppm iron impurities markedly lower OER overpotentials on Ni oxide films [17], and subsequent rigorous electrolyte purification studies demonstrated that removing incidental Fe collapses apparent Ni(OH)_2_/NiOOH OER activity by orders of magnitude, shifting the onset to substantially higher overpotentials [18]. Mechanistic and operando investigations have since clarified that Fe incorporation can create highly active Fe‐centered or Fe‐perturbed sites within (Ni,Fe)OOH, with spectroscopic signatures and activity descriptors consistent with Fe being central to the highest‐activity states (e.g., identification of highly active Fe sites [19], operando analyses of NiFe/Fe oxyhydroxides) [20], yet significant unknowns remain regarding how Fe uptake and speciation couple to electrode microstructure, redox‐accessible active site density, and transport/bubble phenomena in realistic electrodes. From a fabrication and operational engineering perspective, this knowledge gap is consequential: if Fe availability and incorporation kinetics depend on accessible surface area, and/or the spatial arrangement of NiOx features, then “electrolyte Fe effects” cannot be treated as a mere compositional footnote, but must be linked explicitly to how electrodes are manufactured and how their morphology mediates NiOOH/NiFeOOH formation under load [4, 21, 22].

**FIGURE 1:**
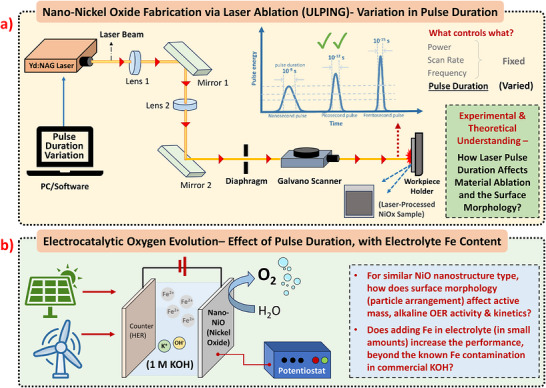
Schematic showing the major concepts explained in this work. (a) The effect of varying pulse duration on nanostructured NiOx fabrication via ULPING, (b) the effect of surface morphology and trace electrolyte Fe content on electrocatalytic OER performance.

Motivated by these coupled challenges, i.e., (i) the need for scalable, binder‐free control of NiOx morphology and loading and (ii) the need to quantitatively connect Fe‐assisted activation to active site density and OER kinetics, this work investigates pulse‐duration‐controlled ultra‐short laser processing (ULPING) as a manufacturing knob to tune nanostructured NiOx electrodes and then interrogates how pulse‐duration‐modulated surface morphology governs active mass utilization, alkaline OER activity/kinetics, and the incremental benefit of adding dilute Fe beyond the background level typically present in commercial KOH. Specifically, we address—(1) how pulse duration alters ablation/oxidation balance and the resulting surface morphology at comparable “nanostructure type”; (2) how the pattern/arrangement of NiOx nanostructures, controls redox‐accessible active site density and kinetic metrics, beyond simply increasing roughness; and (3) whether controlled Fe addition produces performance gains beyond those attributed to incidental Fe, and how such gains scale with morphology‐defined active site availability. The overall objective of the study, and the high‐level methodology, is summarized schematically in Figure 1. Figure 1a outlines nanosecond/picosecond‐level pulsed‐laser fabrication with pulse duration as the primary variable. A systematic electrochemical/kinetic analysis approach is outlined in Figure 1b, wherein baseline (commercial) and Fe‐spiked alkaline electrolytes are compared to directly link fabrication parameters to OER kinetics on Fe‐responsive NiOx surfaces.

## 2 Materials and Methodology

### 2.1 Material Fabrication via ULPING/Laser Processing

High‐purity nickel foils (99.9%, 0.25 mm thick) were used as substrates. Before laser treatment, the foils were sequentially cleaned with acetone, isopropanol, and deionized water, followed by N_2_ drying to eliminate surface contaminants. NiO nanostructures were directly grown on the Ni surface using ultrafast laser ablation (ULPING). Fabrication was carried out with an ultrafast Nd:YAG laser (1064 nm, 1200 kHz repetition rate) under ambient air, while systematically varying the pulse duration. The laser beam, with an effective spot diameter of ∼20 µm, was rastered across the substrate using a galvanometric scanner at controlled scan speeds (15 mm/s) and laser power (10 W), and frequency (1200 kHz). A schematic of the laser fabrication setup is presented in Figure 1a. Our choice of these parameter values (power and scan speed) was based on our earlier investigations that focused on the effect of scan speed and power for electrocatalytic HER/OER, supercapacitive energy storage, etc., as well as other works in similar directions (see Table S1 for comparison) [8, 11, 23, 24, 25, 26]. Pulse duration, being one of the fundamental ablation parameters (linked to ablation physics) rather than a manufacturing/optimization parameter, was studied as an extension to prior works.

A specific Ni sheet was taken for laser processing, and 1 cm x 1 cm pieces were laser processed to obtain the required samples. The 1 cm x 1 cm samples were cut to precise dimensions (slightly bigger on the top for electrode connections) to ensure that every sample is of 1cm^2^ geometric area. (see Figure S1). This was used for electrochemical reactions by connecting the electrodes. Insulating tape was used to mask the non‐lasered area and the blank Ni area on the back.

### 2.2 Structural and Material Characterization

Surface and cross‐sectional morphologies were characterized by field‐emission SEM operated at 5 kV, with magnifications of 10×, 50×, and 1000× to capture features across length scales. Elemental composition and spatial homogeneity were examined using EDX at 15 kV to confirm Ni/O/C stoichiometry and carbon distribution. High‐resolution XPS spectra of Ni 2p and O 1s were acquired using Al Kα radiation (1486.6 eV), with binding energies calibrated to the C 1s peak at 284.8 eV; oxidation states, satellite features, and surface hydroxyl species were resolved using Shirley background subtraction. Crystallographic phases were analyzed by XRD, while surface roughness and three‐dimensional topography were assessed using a profilometer.

### 2.3 Electrochemical Characterization and Analysis

Electrochemical tests were performed in 1.0 m KOH using a three‐electrode cell (NiO working electrode, Pt counter, Hg/HgO reference) and a Biologic (SP‐150) potentiostat.

(1) $$E_{\left(\mathrm{vs}\;\mathrm{RHE}\right)}=E_{\left(\mathrm{vs}\;\mathrm{ref}.\right)}\;\;+0.0591\times \mathrm{pH}+Eo_{\left(\mathrm{of}\;\mathrm{ref}.\right)}$$

The electrochemically active surface area (ECSA) was estimated from the double‐layer capacitance (Cdl) obtained via EIS or CV, assuming a specific capacitance (Cs) of 0.04 mF for planar NiO in alkaline media (ECSA = Cdl/Cs). ECSA‐normalized current density (j_ECSA_ = j_geo_/ECSA) and mass‐normalized current density (j_m_ = j/_mNi_), where _m_Ni is the electroactive Ni mass derived from the Ni^2^^+^/Ni^3^^+^ redox charge, were used to evaluate intrinsic activity. Long‐term stability was assessed by chronopotentiometry at a constant current density of 50 mA cm^−^^2^ for 25 h, tracking the evolution of overpotential with time. For the regular electrochemical evaluation in 1 m KOH, we use Potassium hydroxide ACS reagent, ≥85%, pellets from Sigma–Aldrich. Supplier report indicates Iron (Fe) < 0.001%. For enriching of electrolyte with Fe, Iron(II) sulfate heptahydrate ReagentPlus, ≥99% was used from Sigma–Aldrich. 0.1 g of FeSO_4_ was added to 80 mL of Distilled Water to make a stock solution. 5 mL of this was added to 1 m KOH electrolyte to have a resulting concentration of ∼ 0.4 mm Fe in the electrolytic solution for all the tests, consistent with prior literature suggesting that very trace amount of Fe (e.g. 0.9 ppm—1 mm Fe) is sufficient to induce a boost in catalytic performance [27].

## 3 Results and Discussion

### 3.1 Material Characterization

**FIGURE 2:**
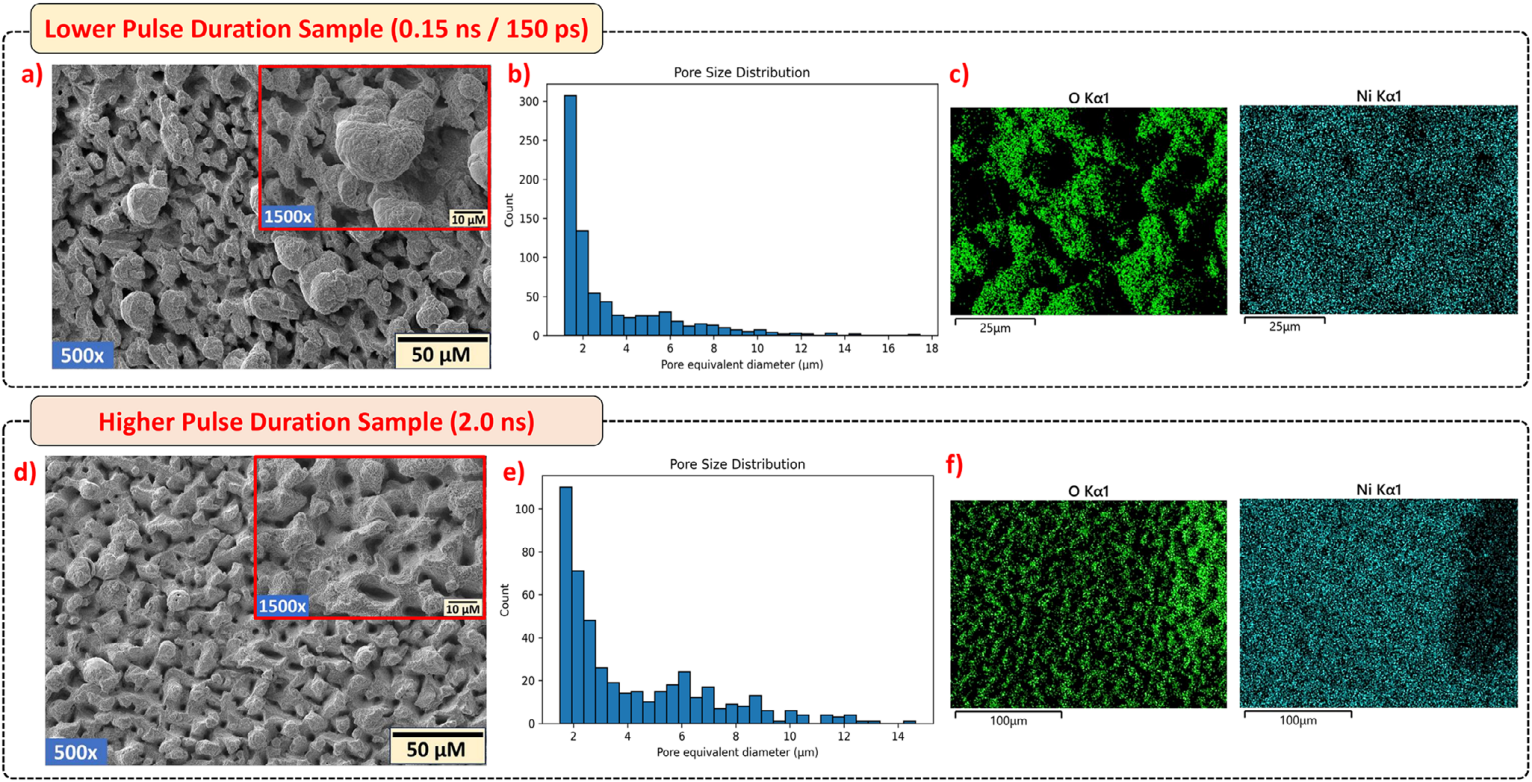
SEM and EDX images of two Pulse Durations showing the nanostructure formation, with porosity analysis. For 0.15 ns, (a) SEM image (scale—50 µm) showing broccoli‐type nanostructures with rough morphology, inset showing—zoomed image at 10 µm; (b) pore size distribution for the sample; (c) EDX image confirming presence of O and Ni. Similarly, for 2 ns, (d) SEM image (scale—50 µm) showing smoother morphology with bigger pore formations, inset showing—zoomed image at 10 µm; (e) pore size distribution analysis for the sample; (f) EDX image confirming presence of O and Ni.

The nanostructural growth and elemental composition of the as‐fabricated materials are done via Scanning Electron Microscopy (SEM) and Energy‐Dispersive x‐Ray Spectroscopy (EDX). In this work, Figure 2 provides a side‐by‐side comparison of how laser pulse duration governs the surface morphology, porosity statistics, and elemental distribution of laser‐fabricated NiOx electrodes, using representative cases of a lower pulse duration (0.15 ns / 150 ps) and a higher pulse duration (2.0 ns). Figure 2a–c corresponds to the 150 ps samples, while Figure 2d–f corresponds to the 2 ns condition, enabling a direct visual and quantitative mapping between pulse‐duration‐controlled nanostructuring and the resultant hierarchical pore–particle architecture that ultimately dictates catalyst accessibility and OER‐relevant interfacial transport. At the macroscale field‐of‐view (500×, scale—50 µm), both electrodes display a continuous laser‐processed layer rather than isolated islands, indicating that the scanning/overlap strategy yields spatially coherent coverage across the imaged region. However, the textural “signature” differs strongly with pulse duration. As in Figure 2a, under 150 ps, the surface exhibits a highly rugged, multiscale morphology composed of flowery, broccoli‐like agglomerates distributed over an interconnected porous backbone. The inset at 1500× (10 µm scale) resolves these agglomerates as rough, nodular clusters with abundant asperities and fine crevices, consistent with rapid energy deposition and constrained thermal diffusion that favors localized ablation, resolidified nano‐/micro‐particulate redeposition, and non‐equilibrium oxidation. In contrast, the 2 ns sample, in Figure 2d, appears smoother and more coalesced, with pore walls that look thicker and less “spiky,” and with pore openings that are visually larger and more rounded. The 1500× inset suggests a morphology dominated by broader, melt‐influenced features with fewer sharp nanoscale protrusions, implying a larger role of thermal accumulation and melt‐mediated mass redistribution at longer pulse durations. This qualitative change is important because it hints that pulse duration is not merely changing “roughness,” but is altering the connectivity and curvature of the solid network that controls electrolyte wetting, bubble pinning/detachment, and the density of redox‐accessible Ni sites that can transform into active NiOOH/NiFeOOH motifs during OER.

The porosity analysis quantitatively reinforces these morphological contrasts by reporting the pore equivalent diameter distribution extracted from image segmentation. For the 150 ps electrode, as shown in Figure 2b, the histogram shows a pronounced population of small pores, with a dominant peak near the lower end of the plotted range (∼ 1–2 µm equivalent diameter) and a long, decaying tail extending up to ∼18 µm. The large counts at the smallest diameters indicate that the 150 ps condition generates a high number density of micro‐pores, consistent with a finely perforated scaffold. Such a distribution is typically advantageous for maximizing geometric surface area, but it can also introduce tighter tortuosity and potentially stronger capillary trapping, which may influence local OH^−^ transport and gas evacuation at high current densities. For the 2 ns electrode shown in Figure 2e, the distribution remains left‐skewed but becomes broader and shifted toward larger pores, with appreciable counts spanning ∼ 2–10+ µm and a maximum pore size closer to ∼14–15 µm in the plotted field. The reduced peak counts (relative to 150 ps) suggest fewer pores overall in the analyzed area, but with larger characteristic pore openings, implying improved permeability pathways through the layer. Collectively, these distributions indicate a pulse‐duration‐driven trade‐off between (i) high micro‐porosity and roughness (150 ps) and (ii) larger through‐pores and smoother walls (2 ns). Such a trade‐off that is central to understanding how nanostructuring impacts OER kinetics under realistic operating regimes where both active‐site density and mass transport/bubble management can be performance‐limiting.

The EDX elemental maps confirm that both morphologies are indeed oxide‐bearing Ni architectures and that the laser‐processed regions contain the expected spatial signatures of Ni and O. In Figure 2c, i.e., for 150 ps, the O Kα1 map, shown in green, is spatially heterogeneous and appears more intense over the rough, porous domains, consistent with oxygen enrichment at nanostructured features and/or increased oxide thickness where surface area and defect density are highest. The Ni Kα1 map (cyan) is comparatively uniform across the imaged region, as expected for a continuous Ni‐based substrate/coating beneath the oxide layer. In Figure 2f, i.e., 2 ns, both O and Ni maps remain broadly co‐localized with the processed surface, but the O signal appears more evenly distributed at the larger mapping scale, consistent with a more continuous oxide coverage over smoother, melt‐influenced topography. More than validating the composition, the EDX mapping supports the key mechanistic inference that the pulse duration modulates not only the pore architecture but also the spatial uniformity of oxidation, which can influence local formation of NiOOH/NiFeOOH under anodic bias and therefore the apparent kinetic response during OER. In the context of this work's objectives, Figure 2 thus establishes a morphological and chemical baseline for interpreting how pulse‐duration‐controlled laser processing tunes accessible active mass and the transport‐relevant pore network, which together control the measured OER kinetics in subsequent electrochemical sections [15, 28].

To understand the surface composition of the as‐fabricated samples, as well as to know the crystalline composition, XPS and XRD are used, respectively. This should help us establish that the effect of pulse‐duration (PD)‐controlled laser processing modulates the oxidation chemistry and phase constitution of the nanostructured NiOx electrodes.

**FIGURE 3:**
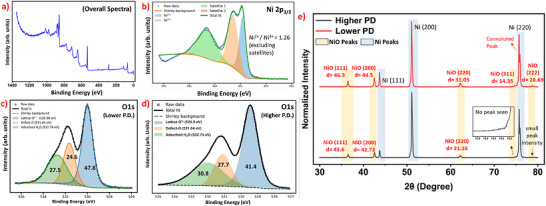
X‐Ray Photoelectron Spectroscopy (XPS) analysis showing the oxidation state analysis for higher and lower PD samples. (a) Overall XPS spectra of Nickel Oxide sample (lower PD). (b) Ni 2p_3/2_ spectra of the lower PD sample, along with the Ni^3+^/Ni^2+^ ratio. (c) O1s spectra of lower PD. (d) O1s spectra of the higher PD sample. (e) x‐Ray Diffraction Spectroscopy (XRD) analysis of higher PD and lower PD showing the presence of different crystalline phases of Ni and NiO, with respective crystallite sizes.

Figure 3a shows the survey/overall XPS spectrum for the lower‐PD NiOx sample. It confirms the expected elemental fingerprint of Ni‐based oxides, with prominent Ni core‐level features (e.g., Ni 2p region) and a clear O 1s signal, indicating that the laser‐processed surface is oxygenated rather than purely metallic. Figure 3b presents a high‐resolution Ni 2p_3_/_2_ spectrum of the lower‐PD sample with a deconvolution comprising Ni^2^^+^, Ni^3^^+^, and two shake‐up satellite contributions (Shirley background fitting). Such satellite‐rich line shapes are characteristic of nickel oxides/oxyhydroxides and arise from strong final‐state and charge‐transfer/multiplet effects rather than simple single‐peak chemical shifts, hence the need for multi‐component fitting rather than assigning oxidation state from a single binding energy position. Foundational analyses of Ni 2p spectral interpretation emphasize precisely these complexities and provide a validated basis for separating Ni^2^^+^/Ni^3^^+^ main‐line and satellite intensity in NiO/Ni(OH)_2_/NiOOH systems [29]. Notably, the figure reports a Ni^2^^+^/Ni^3^^+^ ratio of ∼1.26 (excluding satellites), indicating a mixed‐valence surface with a substantial high‐valent Ni fraction. For OER‐relevant Ni catalysts, this is important because Ni^3^^+^‐like character is often associated with pre‐formed or readily generated oxyhydroxide states (NiOOH‐like motifs) under anodic polarization, and the ability to access these states at lower energetic cost can correlate with improved OER kinetics, especially in Fe‐containing alkaline electrolytes where Ni sites can host Fe incorporation and evolve toward NiFeOOH active motifs [18].

Again, Figure 3c,d compares the O 1s region for lower‐PD and higher‐PD samples, respectively, using a three‐component model consisting of lattice O^2^^−^, defect‐associated oxygen (Defect‐O), and adsorbed H_2_O / hydroxylated surface species. In the lower‐PD sample, the fitted areas (shown within each component) indicate a dominant lattice O^2^^−^ contribution (∼47.8%), accompanied by sizable defect‐O (∼24.6%) and adsorbate‐related oxygen (∼27.5%). In the higher‐PD sample, the lattice fraction decreases (∼41.4%) while the adsorbed component increases (∼30.8%), with defect‐O remaining substantial (∼27.7%). This evolution is consistent with a PD‐driven shift in the near‐surface oxygen environment, and reinstates that shorter pulses often favor rapid quenching and high defect densities (vacancies, under‐coordinated oxygen, grain boundaries), whereas longer pulses can promote greater thermal relaxation, restructuring, and increased hydroxylation/adsorbate stabilization due to altered roughness/porosity and surface energy. In OER electrocatalysis, these distinctions, such as hydroxylated environments or defects in samples, are often important as they could influence OH^−^ adsorption energetics, ease of forming high‐valent metal–oxygen intermediates, the pathway for oxygen evolution etc.

Figure 3e further overlays XRD patterns for the lower‐PD and higher‐PD electrodes, annotating reflections attributable to crystalline NiO (e.g., (111), (200), (220), (311), (222)) and metallic Ni features, from the underlying foil/substrate or partially oxidized regions. The presence of both phases is expected for laser‐processed Ni, where oxidation is spatially heterogeneous through depth: a surface oxide layer forms while a metallic backbone persists, providing electronic conductivity. The figure highlights the NiO peaks and indicates PD‐dependent crystallite size estimates (via peak breadth/Scherrer‐type treatment). One region is explicitly noted as a convoluted peak, emphasizing that peak overlap and mixed phases must be handled carefully when extracting crystallite size. Such phase‐resolved structural information complements the XPS results. The XPS established that the outermost surface is mixed‐valence NiOx with substantial defect/adsorbate oxygen, whereas XRD confirms that the electrode retains a crystalline Ni/NiO framework that can support mechanical robustness and charge transport. PD‐controlled laser processing not only changes morphology (Figure 2) but also measurably shifts surface redox chemistry (Ni^2^^+^/Ni^3^^+^ balance, oxygen defect/hydroxylation signatures) and bulk phase constitution, both of which are tightly coupled to alkaline OER activation pathways and Fe‐assisted catalytic kinetics [30, 31]. Taken together, this figure helps establish (i) the chemical identity of the laser‐grown oxide layer, (ii) the distribution of Ni oxidation states that underpin electrochemical activation into NiOOH/NiFeOOH under OER bias, and (iii) the coexistence of NiO and metallic Ni phases with PD‐dependent crystallite characteristics that reflect distinct laser–matter interaction regimes (ultrashort vs. longer thermal pulses).

### 3.2 Mathematical Modelling of Laser Fabrication

**FIGURE 4:**
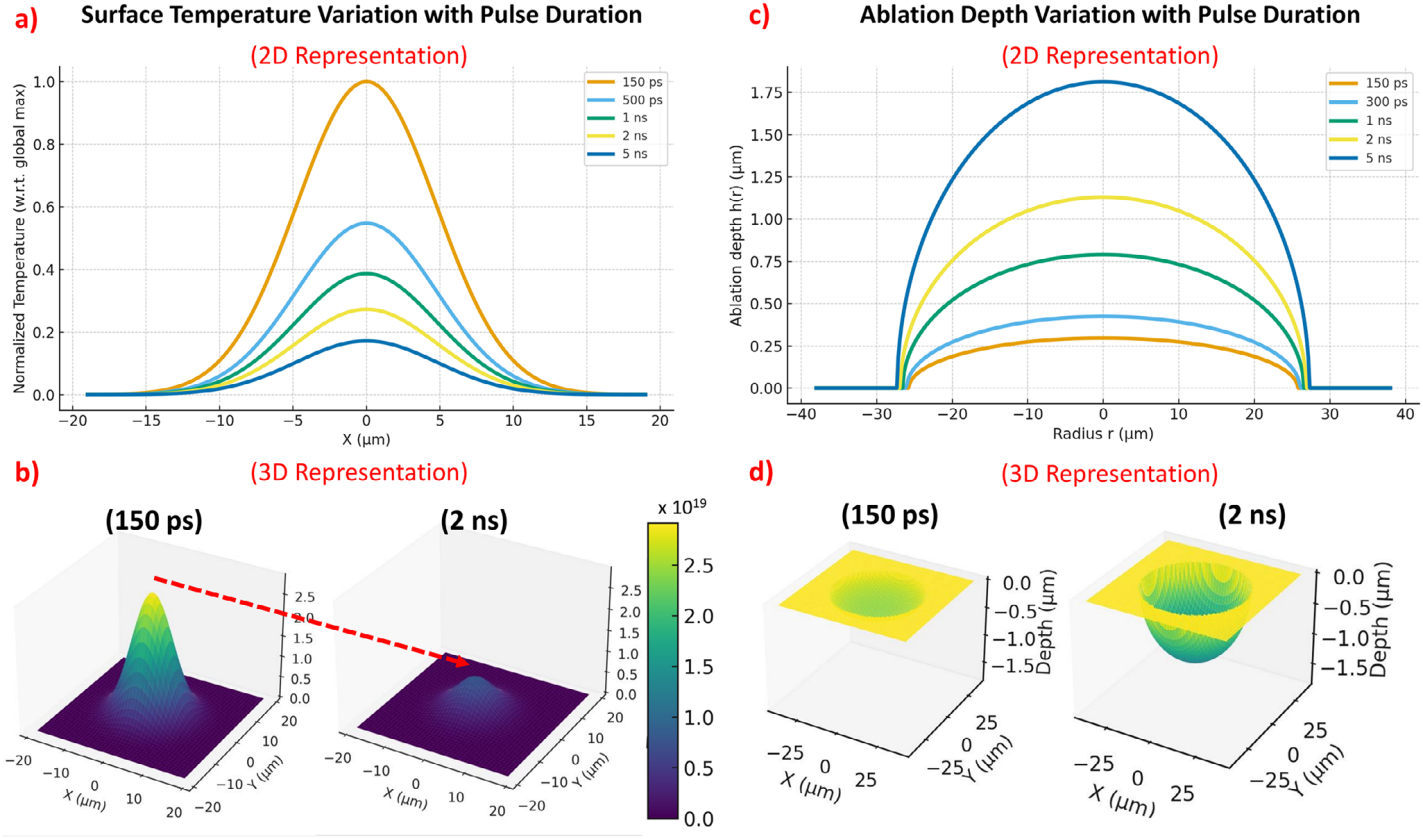
Effect of varying pulse duration on surface temperature and ablation depth via mathematical modelling. (a) Normalized temperature (2D representation) as a function of surface radius for pulse duration 0.15 ns (150 ps)—5 ns. (b) 3D representation of temperature gradient across surface for 150 ps and 2 ns. (c) Variation in ablation depth (2D representation) across surface with changing pulse duration (0.15 ns—5 ns). (d) 3D representation of ablation depth variation across the surface for 150 ps and 5 ns.

Figure 4 presents a physics‐based modeling framework used to rationalize how laser pulse duration controls two coupled outcomes of ultra‐short pulse laser interaction with Nickel: (i) the transient surface temperature field (for melt/oxidation propensity and defect generation) and (ii) the spatially resolved ablation depth profile (a proxy for crater formation, roughness/porosity development, and generation of accessible “fresh” surface). Beyond the two PD variations, 150 ps and 2 ns studied experimentally, the model includes multiple PDs from 150 ps to 5 ns (150 ps, 500 ps, 1 ns, 2 ns, and 5 ns). The paired 2D line representations and 3D surface maps have been presented together to establish an intuitive understanding of the “cross‐sectional” trends with the full radial symmetry of the laser spot. This is directly relevant to electrode/electrocatalyst manufacturing because the temperature field dictates the extent of oxidation, resolidification, and defect formation, while the ablation profile dictates the magnitude and topology of micro‐/mesoscale texturing that ultimately modulates active mass loading and electrolyte/bubble transport during OER.

The spatial temperature distribution induced by nanosecond laser pulses on the nickel surface, we use a physically derived model that accounts for heat conduction, laser absorption, and Gaussian spatial energy distribution. This is based on earlier‐derived works. The resulting temperature T(x,y) at any point on the 2D surface is given by:

(2) $$T_{\left(x,y\right)}=\frac{I_{max}*\;\left(1-R\right)*\sqrt{k}*e^{-\left(\frac{x^{2}+y^{2}}{4k\tau +0.5W^{2}}\right)}}{\sqrt{\pi }*\;K*\;\sqrt{\tau }*\left(1+\frac{8k\tau }{W^{2}}\right)}\;$$

Here, x and y are spatial coordinates (in meters) relative to the laser beam center. W is the laser beam radius (spot size), tau is the laser pulse duration, and R is the reflectivity of the material, which governs how much laser energy is absorbed (gamma = 1–R). The thermal properties of the material—diffusivity k (in m^2^/s) and thermal conductivity K (in W/m·K)—define how quickly heat spreads and how readily the material conducts it. The term I_max_ is the peak laser intensity, calculated from the peak power and beam area. The exponential factor captures the Gaussian decay of temperature away from the beam center due to radial diffusion. This equation enables a full 2D map of the surface temperature to be visualized as a 3D surface plot for various laser powers. Similarly, ablation depth, nanostructure growth height and nano area gain were estimated (semi‐quantitatively) as a function of pulse duration to show possible trends. (see Note S2).

Figure 4a represents the normalized temperature profile along the surface coordinate (X, µm) for pulse durations spanning 150 ps to 5 ns. The curves exhibit a Gaussian‐like spatial envelope centered at the beam axis (X ≈ 0), consistent with a Gaussian beam intensity distribution. A key outcome is that shorter pulses (e.g., 150 ps) produce a sharper, higher‐amplitude peak in the normalized temperature field, whereas longer pulses progressively reduce the peak magnitude and broaden the profile. Physically, this trend indicates that ultrashort pulses deposit energy over a timescale short compared with characteristic heat diffusion and relaxation pathways, producing a more localized and intense near‐surface thermal spike. In contrast, as pulse duration increases into the nanosecond regime, energy deposition overlaps more strongly with heat conduction and phase‐change relaxation, effectively redistributing energy over a larger volume and reducing the maximum surface temperature reached at the hottest point. Such pulse‐duration dependence is consistent with the broader ultrashort‐laser literature, where two‐temperature or related multi‐temperature descriptions are commonly used to capture electron–lattice nonequilibrium during sub‐ns irradiation and the transition toward more thermally mediated behavior at longer durations [32]. Some of our previous reports describe the effect of pulse duration on laser structuring of Ni electrodes (for electrochemical, supercapacitor‐type applications) and also share insights on the effect of laser power and PD on electrocatalytic performance [12, 14]. Figure 4b translates this concept into a full 3D temperature landscape for two representative cases (150 ps and 2 ns). The 150 ps map shows a pronounced “thermal hill” with a steep gradient from the center outward, while the 2 ns map is comparatively flatter and lower in peak height. The strong radial gradient in the shorter‐pulse case implies larger local variations in melt fraction and oxidation kinetics within a single spot, which can seed heterogeneous nucleation, high defect densities, and rapid re‐solidification features—surface attributes frequently linked to increased redox‐accessible sites and altered adsorption energetics in Ni‐based OER electrodes.

Figure 4c,d focus on the second outcome, i.e., the ablation depth profile, plotted as ablation depth (h_(r)_) vs. radius for the same pulse‐duration series, and then visualized as representative 3D crater geometries for 150 ps and 5 ns. The 2D curves in Figure 4c show that, within the model assumptions used here, ablation depth increases systematically with pulse duration. The 150 ps condition yields the shallowest crater, while 5 ns yields the deepest excavation, with intermediate durations (300–500 ps, 1–2 ns) forming a monotonic progression. The crater shapes are radially symmetric “bowl‐like” profiles, with ablation confined primarily within a finite radius defined by the beam footprint and threshold criteria. The accompanying 3D renderings in Figure 4d make this contrast visually explicit and indicate that the 150 ps crater is shallow and gently curved, while the 5 ns crater is markedly deeper with steeper walls and greater volumetric removal.

From a manufacturing standpoint, this result implies that longer pulses can be more effective at generating macroscopically deeper texture (micro‐pits/craters) and higher removed volume per pulse under otherwise comparable beam geometry. These features can increase permeability and create larger through‐pores, consistent with experimentally observed “larger pore” morphologies often associated with greater thermal involvement. Meanwhile, the shorter pulse case, despite achieving higher peak surface temperatures, as in Figure 4a,b, can favor surface‐localized modification (melting/oxidation or defect creation) with comparatively limited net excavation depth, which is a plausible pathway to producing highly roughened, defect‐rich nano‐oxide layers without excessive loss of structural integrity. Temperature localization can influence the fraction of Ni present as Ni^2^^+^/Ni^3^^+^, like surface species and defect oxygen environments that later govern electrochemical activation into NiOOH/NiFeOOH. Simultaneously, the ablation depth/topography dictates the extent of hierarchical texturing that controls wetting, OH^−^ access, and bubble detachment, critical determinants of apparent OER kinetics at high current density [5]. This also provides a framework for interpreting the experimentally observed morphology, active‐site density, and OER kinetic trends in subsequent figures [32, 33, 34].

**FIGURE 5:**
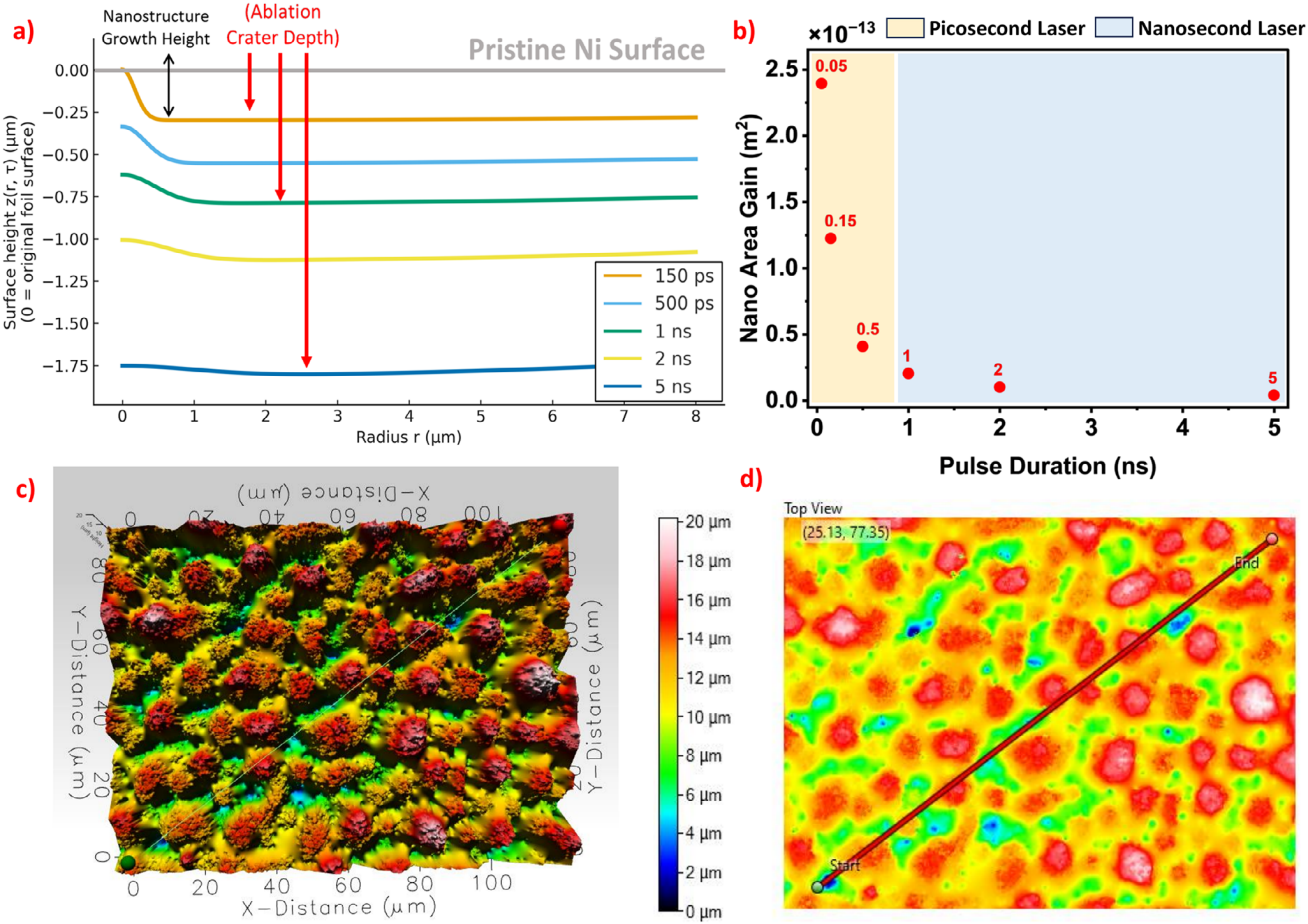
Mathematical modelling and profilometry analysis describing the nanostructure growth. (a) Nanostructure growth height (estimated), along with ‘ablation crater depth’ considered, with pristine Ni surface also marked. (b) Gain in nanostructure area (Nano Area Gain) with varying pulse duration (smaller and larger pulse durations are categorized as nanosecond‐level and picosecond‐level. (c) Profilometry image showing the growth of nanostructures of varying heights in a patterned manner. (d) 2D heatmap showing the nanostructures' growth across the surface, with particular spots showing extremely tall growth. The direction of analysis is diagonal (start and end shown).

Though the previous model talks about surface temperature and foil (Ni) ablation, the growth of nanostructure, driven by ablation and the subsequent processes, can also be modelled in a phenomenological/empirical manner and give a good idea of the nanofabrication process. Figure 5 integrates physics‐based modeling with experimental surface profilometry to elucidate how pulse‐duration‐controlled laser processing governs the net nanostructure growth, the balance between material removal (ablation) and material buildup (oxide/nanostructure formation), and the resulting spatial heterogeneity across the Ni surface. Unlike Figures 2, 3, 4, which separately addressed morphology, chemistry, and transient laser–matter interaction, Figure 5 explicitly links these phenomena into a net topographical outcome, which is the parameter most directly relevant to electrochemical performance because it dictates true accessible surface area, active mass distribution, and local transport environments during OER.

Figure 5a presents a radial height profile derived from the mathematical model, plotting the surface height Z_(r, tau)_ relative to the pristine Ni surface (Z = 0) as a function of radius for pulse durations ranging from 150 ps to 5 ns. Two competing processes are highlighted here: (i) nanostructure growth height, indicated by the upward displacement relative to the crater bottom, and (ii) ablation crater depth, marked by red arrows that quantify the extent of net material removal below the original surface. The curves clearly show that increasing pulse duration monotonically increases the absolute crater depth, from a shallow depression at 150 ps to a much deeper excavation at 5 ns, consistent with the ablation‐depth trends predicted in Figure 4. At the same time, the vertical separation between the pristine surface and the modeled nanostructured surface reflects the effective growth height of laser‐induced oxide/nanostructures. Notably, shorter pulse durations preserve the surface closer to the pristine plane while still generating significant roughness, whereas longer pulses push the system toward a regime dominated by material removal. This distinction is critical for electrocatalysis: excessive ablation can reduce catalyst utilization efficiency by removing conductive backbone material, while insufficient ablation may limit pore connectivity and transport. The modeling thus rationalizes why an intermediate‐to‐short pulse duration window is often optimal for generating high‐surface‐area, binder‐free electrodes without sacrificing mechanical or electrical continuity.

Further, Figure 5b abstracts these geometric effects into a scalar metric termed ‘Nano Area Gain’, which quantifies the increase in effective nanostructured surface area relative to a flat reference. Pulse durations are grouped into picosecond‐level (0.05–0.5 ns) and nanosecond‐level (1–5 ns) regimes. The data reveals a non‐linear dependence, i.e. the nano‐area gain increases sharply as pulse duration increases from ultrashort picosecond values, reaches a maximum in the sub‐nanosecond to ∼1 ns range, and then diminishes at longer nanosecond durations. This trend captures the essential trade‐off between surface amplification (via nanostructure growth and moderate crater formation) and surface loss/smoothing (via excessive melting and deep ablation). From an OER perspective, this metric is particularly powerful because electrochemical current density scales not simply with geometric area but with electrochemically active surface area (ECSA), which itself is strongly influenced by hierarchical roughness and porosity. Similar non‐monotonic relationships between roughness, porosity, and activity have been reported in laser‐textured and electrochemically roughened Ni‐based OER electrodes, where optimal performance arises from a balance between site density and transport accessibility.

Figure 5c provides 3D optical profilometry data that experimentally validate the modeling predictions. The rendered surface map shows a periodic, patterned distribution of nanostructures with heights spanning several micrometers, arranged according to the laser scanning path. The color contrast reveals pronounced topographical modulation: raised “islands” or agglomerates are interspersed with lower valleys, creating a multiscale roughness landscape. Such patterned growth is characteristic of pulsed laser processing, where overlapping Gaussian beam footprints and repeated thermal cycling lead to preferential redeposition and growth at specific loci. Importantly, the magnitude of height variation observed here is consistent with the modeled growth heights and crater depths in Figure 5a, lending confidence to the combined modeling–experimental framework. For electrocatalysis, these height variations imply spatially varying local current density, bubble nucleation propensity, and electrolyte access. Figure 5d further analyzes the profilometry data via a 2D height heatmap, with a diagonal line marking the direction of a representative line scan. The heatmap emphasizes that while the surface is globally nanostructured, localized “hot spots” of exceptionally tall growth (red/white regions) coexist with lower‐lying regions (blue/green). Such heterogeneity is not merely a processing artifact; it can be advantageous by creating a distribution of microenvironments that support different aspects of OER operation—high‐curvature regions can stabilize high‐valent NiOOH/NiFeOOH species, while deeper valleys can serve as channels for electrolyte replenishment and bubble escape. Recent studies on NiFe oxyhydroxide electrocatalysts highlight that spatial heterogeneity in oxidation state and coordination environment can enhance apparent activity by broadening the population of active motifs. A quantitative and visual link between pulse‐duration‐controlled laser physics and the net nanostructured topography that governs electrocatalytic function is established in Figure 5, overall. By showing how pulse duration simultaneously controls ablation depth, growth height, surface area gain, and spatial heterogeneity, the figure provides a mechanistic basis for correlating fabrication parameters with ECSA, active‐site density, and ultimately OER kinetics in binder‐free NiOx electrodes.

### 3.3 Evaluation of Electrocatalytic Performance

**FIGURE 6:**
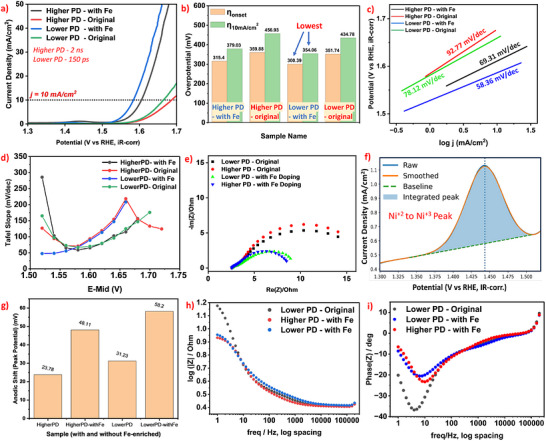
Electrocatalytic activity, kinetics, and charge‐transfer analysis for Oxygen Evolution Reaction (OER). (a) Linear Sweep Voltammetry (LSV) of two samples, with and without Fe. (b) Overpotential values for onset and j = 10 mA/cm^2^. (c) Tafel analysis with slope of the linear‐fitted region shown for all the samples. (d) Value of tafel slope by varying the potential region chosen for analysis. (e) Nqyuist plot (EIS) for all samples. (f) Oxidation peak (sample: Lower PD—Original) from LSV (5 mV/s) for Ni^2+^ to Ni^3+^ (g) Anodic shift in Ni oxidation peak potential (h) Bode plot showing variation of |Z| with frequency. (i) Bode plot showing variation of phase (Z) with frequency.

Figure 6 consolidates the electrochemical performance, kinetic descriptors, and charge‐transfer characteristics of pulse‐duration‐engineered NiOx electrodes, explicitly contrasting lower pulse duration (150 ps) and higher pulse duration (2 ns) samples, both in their as‐prepared (original) state and after Fe incorporation from electrolyte. The multi‐panel presentation systematically links macroscopic activity metrics (LSV, overpotential), kinetic analysis (Tafel slopes), and interfacial charge‐transport behavior (EIS, Bode plots) to microscopic indicators of active‐site density (Ni^2^^+^/Ni^3^^+^ redox charge), thereby offering a coherent picture of how fabrication parameters and electrolyte chemistry co‐regulate OER performance. Figure 6a shows linear sweep voltammetry (LSV) curves recorded in alkaline electrolyte (1 m KOH), highlighting the clear activity enhancement associated with both lower pulse duration nanostructuring and Fe presence. The lower‐PD electrode exhibits an earlier OER onset and steeper current rise compared to the higher‐PD counterpart, indicating intrinsically more favorable kinetics. Upon Fe addition, both electrodes show further leftward shifts of the polarization curves, but the improvement is most pronounced for the lower‐PD sample, underscoring a synergistic interaction between morphology‐induced active site density and Fe‐assisted catalytic pathways. This behavior is consistent with extensive literature demonstrating that NiOOH alone is moderately active, whereas incorporation of trace Fe converts the active phase into highly efficient NiFeOOH, dramatically reducing the kinetic barrier for OER [5, 18].

Figure 6b quantitatively summarizes the overpotential values extracted at both onset and at a benchmark current density of 10 mA cm^−^^2^, a widely accepted metric for comparing alkaline OER catalysts. The bar chart clearly shows that the lower‐PD + Fe sample exhibits the lowest overpotential, followed by lower‐PD original, higher‐PD + Fe, and finally higher‐PD original. This ordering directly reflects the combined effects of (i) higher nano‐area gain and accessible active mass for the lower‐PD electrode (Figures 2 and 5) and (ii) Fe‐enabled acceleration of the rate‐determining O─O bond formation step in Ni‐based oxyhydroxides. Importantly, the fact that Fe addition yields diminishing returns for the higher‐PD sample suggests that morphology‐limited active site availability can cap the benefit of Fe incorporation, emphasizing the importance of fabrication‐driven surface engineering. The Tafel plots derived from the kinetic region of the LSVs are shown in Figure 6c, with linear fits indicating the apparent Tafel slopes. The slopes span from ∼92.7 mV dec^−^^1^ for the least active configuration to as low as ∼58.4 mV dec^−^^1^ for the lower‐PD + Fe electrode. Such a reduction in Tafel slope signifies a fundamental change in the OER kinetics, often interpreted as a transition toward a faster proton‐coupled electron transfer step or altered surface coverage of key intermediates (*OH, *O, *OOH). The observed values align well with reported Tafel slopes for NiFeOOH systems under alkaline conditions, where Fe incorporation is known to lower the energetic barrier for *OOH formation. Figure 6d addresses an often‐overlooked but critical issue—the dependence of extracted Tafel slope on the chosen potential window. By plotting Tafel slope as a function of the upper/lower potential bounds, the figure demonstrates that while absolute values vary, the relative ranking of samples remains robust, with the lower‐PD + Fe electrode consistently exhibiting the lowest slope across windows. This analysis strengthens the credibility of the kinetic conclusions and avoids overinterpretation based on a single arbitrarily chosen linear region—a practice increasingly recommended in rigorous electrocatalysis studies.

Fe being one of the main OER active sites, the incorporation of Fe from the Fe‐enriched electrolyte is preferred and begins from the surface rather than at the bulk, as supported by earlier studies [35]. Only with continued cycling or longer restructuring does a larger fraction become more uniformly incorporated into interior substitutional sites [36, 37]. Earlier operando/x‐ray fluorescence work found Fe uptake to occur predominantly at edge sites with higher oxygen‐vacancy density. A recent single‐particle study again identified edge sites as critical in NiFe hydroxide OER behavior [38]. Overall, this suggests that initial Fe incorporation may easily occur at accessible edge/defect sites, forming Ni─O─Fe species that dramatically enhance catalysis, but with minimal effect on the majority of the Ni redox species. A work by Bao et al. gives important insights in this direction by experimentally demonstrating that in unpurified (as‐purchased) KOH, the slight amount of Fe present keeps redepositing and creating a more disordered and defect‐rich structure which is directly linked to OER activity [39]. Raman and Grazing Incidence—XRD (GI‐XRD) show most structural transformations occurring on the surface, during CV cycling, with bulk of the film remaining unchanged.

To note here, however, is that a universal atomic ranking, like vacancy sites are better than steps or terraces, is exactly missing in the literature. Undercoordinated defect‐rich sites are found better than one universally dominant defect. A DFT/AIMD work on NiOOH found that water adsorption/intercalation can lower surface/interface energies enough to permit Fe adsorption not only on the outer surface but also on inner layer surfaces of hydrated NiOOH. Some others report the role of local/neighboring sites, such as Fe‐containing local motif lowers the OER overpotential substantially, or Fe helps stabilize a defective surface and modifies the neighboring Ni electronically [40, 41].

Figure 6e shows Nyquist plots (EIS) for all samples, revealing semicircular features associated with the charge‐transfer resistance (Rct) at the electrode–electrolyte interface. The lower‐PD electrodes display smaller semicircle diameters than higher‐PD ones, indicating lower Rct and more facile interfacial electron transfer. Fe addition further suppresses Rct, consistent with the formation of a more conductive and catalytically competent NiFeOOH phase. These trends corroborate the LSV and Tafel results, linking improved kinetics to reduced interfacial impedance rather than merely increased geometric area. Further, Figure 6f focuses on the Ni^2^^+^ → Ni^3^^+^ oxidation peak extracted from low‐scan‐rate (5 mV s^−^^1^) LSV for the lower‐PD original sample. The shaded integrated peak area, obtained after baseline correction, is directly proportional to the electrochemically addressable Ni active site density and NiO mass participating in redox cycling. This metric can be used as a quantitative bridge between morphology (laser‐induced nanostructuring) and electrochemical performance. Further, we track the relative Fe incorporation in two samples by the anodic shift of the oxidation peak potential as shown in Figure 6g. A more positive shift corresponds to the incorporation of Fe from the electrolyte to the bulk (after surface incorporation) and is seen more in the case of Fe‐enriched electrolyte, with lower PD having more incorporation.

Finally, Figure 6h,i presents Bode magnitude (|Z|) and phase angle plots, respectively. The lower‐PD and Fe‐containing samples show reduced impedance magnitude across the frequency range and phase‐angle features indicative of more ideal capacitive behavior and faster charge‐transfer dynamics. The suppression of low‐frequency impedance and shifts in phase minima further support the conclusion that pulse‐duration‐optimized morphology and Fe incorporation jointly enhance interfacial kinetics and charge transport. In summary, Figure 6 establishes a direct, multi‐scale correlation between pulse‐duration‐controlled fabrication, Fe‐assisted surface chemistry, and OER activity/kinetics. The convergence of LSV, Tafel, EIS, and redox‐charge analyses demonstrates that the superior performance of the lower‐PD + Fe electrode arises not from a single factor but from the synergistic optimization of active‐site density, intrinsic reaction kinetics, and interfacial charge‐transfer efficiency [4, 18, 19, 20, 30].

Unlike conventional NiOx catalyst‐layer electrodes, where activity and durability depend strongly on ink composition, binder coverage, and particle‐to‐substrate adhesion, the present ULPING strategy forms NiOx directly from Ni in a one‐step, binder‐free manner and uses pulse duration as a physically meaningful control parameter for defect density, nano‐area gain, ablation depth, and hierarchical porosity. Although some intentionally Fe‐preloaded NiFe/Ni‐foam electrodes report lower overpotentials, those systems are not mechanistically equivalent to electrolyte‐activated NiOx (more comparison is given in Table S2) [42, 43, 44]. Also, Nafion is universally known to alter (sometimes for better or worse) alkaline OER measurements through site coverage, wetting/bubble behavior, and oxidative instability; accordingly, binder‐free NiOx electrodes provide a cleaner platform for Fe‐incorporation studies than binder‐based catalyst layers tested in different (GDE/MEA) configurations. The present platform is therefore particularly valuable for studying OER in Fe‐enriched alkaline media, because it combines direct electrical contact, preserved nanostructural integrity, and a cleaner interface for probing dynamic Fe incorporation without the added uncertainty of polymer‐binder effects.

**FIGURE 7:**
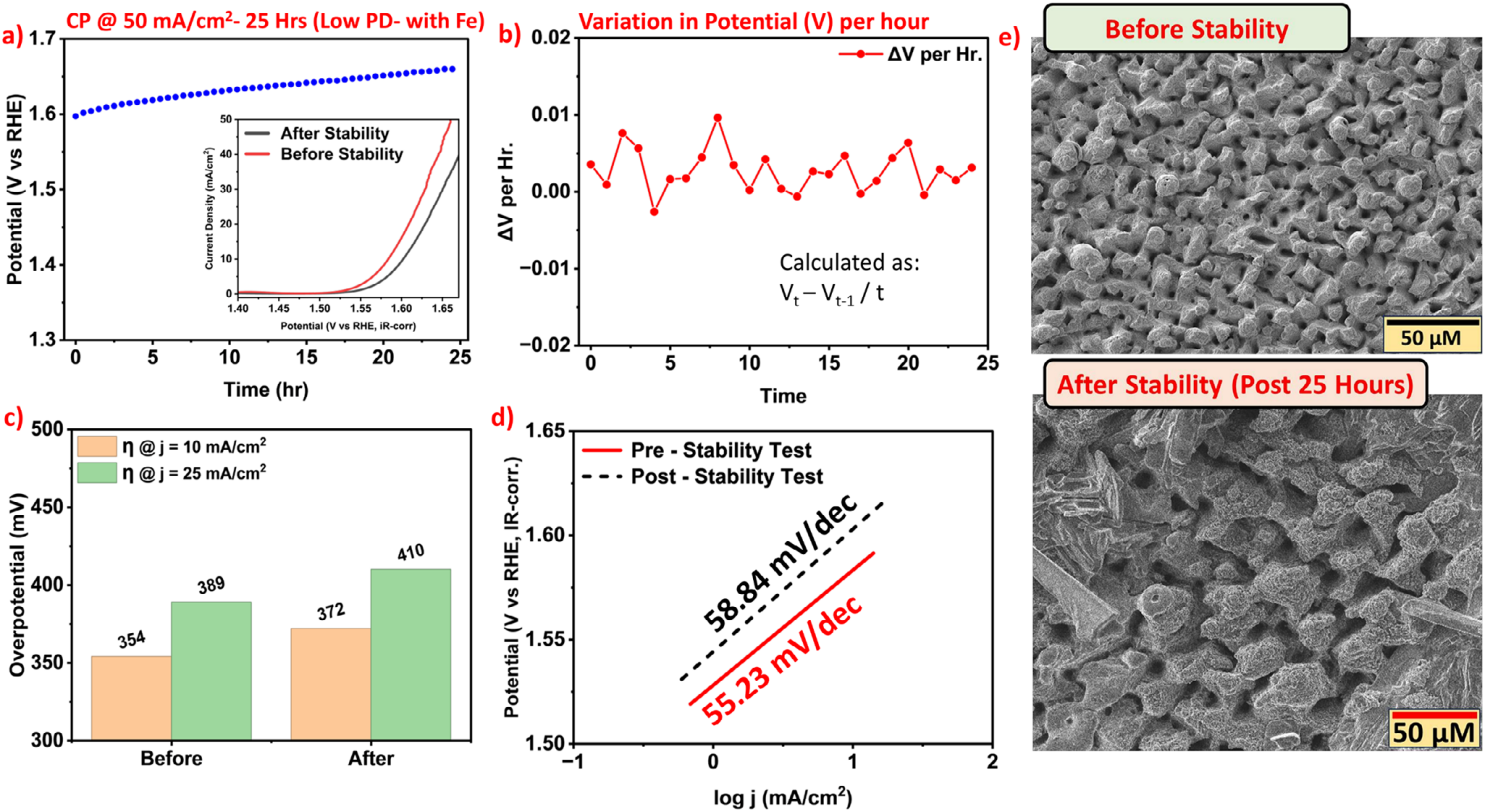
(a) Stability analysis via chronopotentiometry (CP) of ‘Low PD‐ with Fe’ sample at 50 mA/cm^2^ for 25 h. (inset—LSV before and after stability) (b) Hourly variation in potential over time. (c) Bar graph showing change in overpotential (j = 10 mA/cm^2^ & 25 mA/cm^2^), before and after stability analysis. (d) Tafel slope pre‐ and post‐ stability test. (e) SEM image (50 µm) showing a change in nanostructure morphology and film quality.

Figure 7 evaluates the long‐term operational stability of the most active catalyst configuration identified in this study—namely, the low pulse‐duration (Low PD) NiOx electrode operated in Fe‐containing alkaline electrolyte—under industrially relevant current density conditions. Beyond demonstrating sustained activity, the figure correlates electrochemical durability with kinetic retention and post‐operation morphological integrity, which together are critical requirements for practical oxygen evolution reaction (OER) electrodes. Figure 7a presents chronopotentiometry (CP) performed at a constant current density of 50 mA cm^−^^2^ over 25 h, a stress condition significantly more demanding than conventional 10 mA cm^−^^2^ benchmarking. The potential–time trace shows a largely monotonic and gradual increase in operating potential, without abrupt jumps or major instabilities, indicating robust catalytic operation and mechanical adhesion of the laser‐fabricated, binder‐free electrode layer. The absence of sudden potential spikes further suggests stable gas evolution behavior with minimal catalyst delamination or catastrophic bubble‐induced detachment. The inset LSV curves recorded before and after the stability test show only a marginal shift in polarization, confirming that the intrinsic OER activity is largely retained after prolonged anodic operation. Such behavior is notable for Ni‐based catalysts, which often suffer from surface reconstruction, loss of active Fe species, or gradual passivation under sustained bias if the electrode architecture is not mechanically robust or chemically resilient. Figure 7b provides a more granular view of stability by plotting the hourly variation in operating potential over the 25‐h CP test. The data show small, quasi‐random fluctuations around a narrow band, rather than a monotonic drift, suggesting that transient changes, likely associated with bubble coverage, local wetting/dewetting, or minor surface restructuring, are reversible and do not accumulate into irreversible degradation. This behavior is characteristic of stable NiFe oxyhydroxide systems, where the active phase dynamically restructures under OER conditions but remains catalytically competent as long as the underlying support and morphology are preserved.

Figure 7c quantifies durability in terms of overpotential retention, comparing values at 10 and 25 mA cm^−^^2^ before and after the stability test. While a modest increase in overpotential is observed post‐stability, the increase is relatively small compared to the absolute activity gains achieved through pulse‐duration optimization and Fe incorporation (Figure 6). Importantly, the retention of low overpotential at both moderate and higher current densities indicates that neither the active‐site density nor the charge‐transfer pathways are severely compromised during long‐term operation. This is particularly relevant for NiFe‐based OER catalysts, where Fe leaching or redistribution can sometimes lead to significant performance decay if the catalyst–electrode interface is not well engineered. Figure 7d compares the Tafel slope before and after the 25‐h CP test, yielding a post‐stability value of approximately 55.2 mV dec^−^^1^. The near‐invariance of the Tafel slope indicates that the fundamental OER reaction mechanism and rate‐determining step remain unchanged after prolonged operation. In other words, while slight energetic penalties may arise due to surface aging or partial restructuring, the catalyst continues to operate via the same efficient kinetic pathway associated with Fe‐assisted NiOOH/NiFeOOH active sites. Preservation of a low Tafel slope after stability testing is a strong indicator of true catalytic durability, rather than mere apparent stability due to increased surface roughness or oxide thickening.

Figure 7e provides SEM images (50 µm scale) of the electrode surface before and after the stability test, offering direct morphological evidence of structural robustness. Prior to operation, the surface exhibits the characteristic hierarchically porous, broccoli‐type nanostructure generated by low‐PD laser processing. After 25 h at 50 mA cm^−^^2^, the overall porous framework is retained, with no signs of large‐scale cracking, delamination, or catastrophic collapse of the nanostructured layer. Some degree of surface smoothing and localized restructuring is observed, which is expected given the dynamic nature of Ni‐based oxyhydroxides under sustained anodic polarization. Notably, the persistence of the interconnected porous backbone suggests that the laser‐fabricated architecture effectively accommodates repeated gas evolution and electrochemical cycling stresses—an advantage over conventional powder–binder electrodes, where polymer binders often degrade or block active sites during long‐term operation. Taken together, here we demonstrate that the synergy between low pulse‐duration laser nanostructuring and Fe‐assisted activation not only maximizes OER activity and kinetics but also delivers excellent operational stability under demanding conditions. The combination of stable CP response, minimal kinetic degradation, preserved Tafel behavior, and retained morphology underscores the suitability of this binder‐free, laser‐fabricated NiOx electrode for sustained alkaline OER. These findings align with, and extend beyond, prior reports on NiFe oxyhydroxide durability by explicitly showing how manufacturing parameters (pulse duration) can be leveraged to engineer both performance and longevity in earth‐abundant OER catalysts. Further, as a validation of Fe incorporation in NiOx, we provide a deconvoluted XPS of Fe 2p region for a NiOx sample after 25 CV cycles (Figure S2). Ex situ characterization techniques such as XPS or EDX are deemed unreliable for tracking and properly quantifying the presence of Fe and hence more rigorous *operando* and in situ techniques are necessary (see Note S1) [45].

The as‐incorporated Ni(Fe)OOH OER catalyst can also undergo leaching during constant current operation. However, the advantage of using a NiOx pre‐catalyst is manifested here as the required amount of Fe is incorporated from the electrolyte to create an OER‐active catalyst, without requiring a NiFe alloy that is directly prone to Fe dissolution. In this work, the starting NiOx surface transforms into NiOOH or NiOxHy active state, and Fe from the electrolyte is incorporated primarily at the surface or near‐surface, and not in bulk (as discussed earlier). The electrochemical signature of Ni oxidation provides some insights into the transformation to hydroxide and Fe incorporation. Under sustained OER, the Fe population is dynamic as some Fe is retained at the active surface, some can be lost by dissolution/leaching, and some can end up in the subsurface as the Ni oxyhydroxide restructures. By the use of ToF‐SIMS, a recent work proposed that during prolonged electrolysis, Fe can be depleted from the surface and part of the ‘lost’ Fe is found either in the electrolyte or in the sub‐surface region rather than on the active surface [46].

Additionally, dissolution is expected during the hold, and it is better viewed as a quasi‐steady exchange than as a one‐way process. In Fe‐lean conditions (less Fe, as in unpurified KOH), surface Fe is progressively depleted and activity drops. Hence, by the use of Fe‐enriched electrolytes, the surface Fe population can be maintained better because dissolution is partly compensated by redeposition. As described before, the ex situ characterization of Fe content by XPS or EDX is challenging and hence future works should specifically focus on the remaining unanswered questions such as mechanism and extent of Fe incorporation and dissolution [46, 47].

## 4 Conclusion and Future Directions

The central motivation of this work was to address a persistent and underexplored challenge in alkaline oxygen evolution electrocatalysis: how fabrication physics directly governs catalytic kinetics, beyond compositional tuning alone. While Ni‐based (oxy)hydroxides, particularly Fe‐activated NiOOH/NiFeOOH, are widely recognized as state‐of‐the‐art non‐precious OER catalysts, their performance is often discussed independently of the manufacturing pathway used to generate the electrode–catalyst interface. This study demonstrates that pulse‐duration‐controlled laser nanostructuring provides a direct, scalable, and binder‐free route to engineer this interface and, critically, to link laser–matter interaction fundamentals to electrochemical performance descriptors. By systematically varying laser pulse duration from the picosecond to nanosecond regime, we show that pulse duration fundamentally alters the balance between localized energy deposition, thermal diffusion, ablation depth, and nanostructure growth. Mathematical modeling reveals that shorter pulses generate sharper and more intense surface temperature gradients with limited net ablation, favoring defect‐rich oxide growth and high nano‐area gain. In contrast, longer pulses promote deeper crater formation and greater material removal, resulting in smoother morphologies with larger but fewer pores. These predictions are validated experimentally through SEM, pore size analysis, and profilometry, establishing pulse duration as a dominant parameter controlling hierarchical porosity and surface topology.

Electrodes fabricated at low pulse duration exhibit markedly higher redox‐accessible Ni^2^^+^/Ni^3^^+^ charge, lower charge‐transfer resistance, and faster OER kinetics than higher pulse‐duration counterparts. These gains are not purely geometric but reflect a higher density of catalytically competent Ni sites that transform into active oxyhydroxide phases under anodic bias. Upon Fe introduction, either incidentally from alkaline electrolyte or deliberately in controlled amounts, the performance enhancement is most pronounced for the low pulse‐duration electrode. This reveals a central insight that Fe‐assisted OER activity is strongly morphology‐dependent, and Fe's catalytic benefit is maximized only when sufficient active site density and accessible surface area are created by fabrication. Combined XPS, XRD, and electrochemical analyses show that laser‐fabricated NiOx surfaces possess mixed Ni^2^^+^/Ni^3^^+^ character, defect‐associated oxygen, and stable Ni/NiO backbones, facilitating rapid activation into NiFeOOH while preserving conductivity and robustness. Chronopotentiometry at 50 mA cm^−^^2^ for 25 h confirms sustained activity, minimal kinetic degradation, and retained nanostructural integrity, underscoring the durability advantage of binder‐free laser fabrication.

From a broader perspective, this work establishes several key advantages of pulse‐duration‐engineered ULPING for electrocatalyst manufacturing:

- chemical‐free, binder‐free fabrication directly on conductive substrates;
- scalability to centimeter‐scale electrode areas with high repeatability;
- independent control of surface chemistry and morphology through laser physics rather than precursor chemistry; and
- direct coupling between fabrication parameters and kinetic descriptors, enabling rational optimization rather than empirical trial‐and‐error.

Despite these advances, several limitations warrant acknowledgment. First, the laser‐matter interaction model used here offers a simplified description of ultrafast energy transfer and does not fully capture multiphysics effects such as plasma formation, dynamic reflectivity, or transient oxidation kinetics. Second, Fe incorporation was achieved mainly via electrolyte addition rather than Fe loading within the oxide lattice, which may lead to a different understanding of Fe's role in electrocatalytic NiOx activity. Third, although post‐mortem characterization and electrochemical diagnostics support NiFeOOH as the active phase, in situ or operando spectroscopic validation is still needed for definitive mechanistic attribution. Looking forward, integrating multi‐pulse strategies, beam shaping, and dynamic pulse sequencing could enable finer control over porosity and active site distribution. Extending pulse‐duration‐controlled processing to multimetal systems (e.g., Ni─Fe─Co, Ni─Fe─Mn) or metal‐carbon (metal/graphene) systems may allow compositionally graded active phases [48]. Coupling ULPING with operando Raman or XAS and testing in flow‐cell or gas‐diffusion configurations will be essential for industrial relevance.

In summary, this study demonstrates that pulse duration is not merely a laser parameter but a powerful materials design variable, capable of dictating morphology, active site density, and OER kinetics. By unifying laser physics, materials characterization, and electrochemical analysis, this work provides a blueprint for manufacturing next‐generation, earth‐abundant OER electrodes through rational, physics‐guided design [49, 50, 51, 52, 53, 54, 55, 56].

## Author Contributions

The manuscript was written through the contributions of all authors. A.K. and S.K. did the conceptualization. S.K. did the preliminary collection of data, followed by J.R. and S.K. doing the data analysis, writing, and editing, hence they share equal authorship. A.K. supervised the conceptualization, overall writing of the manuscript, and provided expert suggestions. The final version of the manuscript was approved by all authors before submission.

## Funding

This work was partially supported by the Natural Sciences and Engineering Research Council of Canada (NSERC) and Mitacs, Canada.

## Conflicts of Interest

The authors declare no conflicts of interest.

## Supporting information




**Supporting File**: smll73897‐sup‐0001‐SuppMat.docx.

## Data Availability

The data that support the findings of this study are available on request from the corresponding author. The data are not publicly available due to privacy or ethical restrictions.
