# Supplementary material for: Linking Pulse‐Duration‐Controlled Laser Nanostructuring to Oxygen Evolution Kinetics in Fe‐enriched NiOx Electrodes
Source: Small. 2026 May 20;22(38):e73897. doi: 10.1002/smll.73897 (PMC13351486; doi:10.1002/smll.73897)
Supplement: Supplementary file 1 — Supporting File: smll73897‐sup‐0001‐SuppMat.docx. [file SMLL-22-e73897-s001.docx]

*Supporting Information*

**Linking Pulse-Duration-Controlled Laser Nanostructuring to Oxygen Evolution Kinetics in Fe-enriched NiOx Electrodes**

Sandra Susan Koshy, ^#^ ^a, b^ Jyotisman Rath, ^#^ ^c, d^ Amirkianoosh Kiani ^* a, b^

* To whom correspondence should be addressed

# Equal contribution

a - Silicon Hall: Micro/Nano Manufacturing Facility, Ontario Tech University, Oshawa, Ontario L1G 0C5, Canada

b - Department of Mechanical and Manufacturing Engineering, Ontario Tech University, Oshawa, ON L1G 0C5, Canada

c - Department of Chemical Engineering, Institute of Chemical Technology – IndianOil Odisha Campus, Bhubaneswar - 751013, Odisha, India

d - Davidson School of Chemical Engineering, Purdue University, 480 Stadium Mall Drive, West Lafayette, IN 47907, USA

**Table S1**- Choice of laser parameters for fabrication via pulsed laser ablation (ULPING)

| **Laser Parameter** | **Key Points / Usefulness** | **References** |
| --- | --- | --- |
| Power | A control of delivered energy - raising power generally increases oxide growth, roughness, and nanostructuring but enhanced power (beyond 15 or 20 W) can lead to overoxidation and damage to nanostructures. An intermediate power (10 W) ensures a base case for comparison (with optimum around 5W -15 W) | ^[1–4]^ |
| Scan Speed | Controls dwell time and overall fabrication time. Lower scan speed gives the beam more residence time, usually causing more oxidation, rougher / more porous oxide. This study uses 15 mm/s as a base case. Higher scan speeds (50 -100) can prove to be better to have a more perfectly aligned surface with reduced overlap. Speeds up to 500mm/s or more could result in very limited oxide growth (at the benefit of highly reduced fabrication time). | ^[1,2]^ |
| Frequency | Sets pulse accumulation rate. Higher frequency is used to promote faster oxide formation / pulse overlap, whereas lower can alter the ablation dynamics. | ^[1,5]^ |
| Pulse Duration | Crucial surface-machining parameter. Shorter pulses are preferred for finer, near-nano morphology with less collateral thermal/mechanical damage, which is favorable for high-surface-area TMO electrodes. This work explores **150 ps** to **5 ns.** | ^[6]^  This Work |
| Others- Hatching Space, Laser Pattern | This is referred to as pitch, the center-to-center distance between adjacent scan lines. Smaller pitch should increase line overlap and oxide coverage; larger pitch should leave more untreated gaps. Also, a laser pattern (shape) can be set as in simple rectangular layout or circular. | ^[7]^ |

**Table S2**- Brief comparison of NiOx samples from literature with similar research directions

| **System** | **Fabrication / electrode concept** | **Representative OER performance** | **References** |
| --- | --- | --- | --- |
| Binder-free ULPING NiOx on Ni (Pulse Duration Variation) | Direct picosecond laser oxidation/texturing of Ni, no binder or carbon additive, pulse duration used as a primary morphology/defect/porosity knob | η_10_ - 354 mV, η_50_ - 372 mV  Tafel - 55 mV/dec  stable for 25 h at 50 mA cm⁻² | — |
| ULPING NiO on metal foil (prior laser route) | Direct ultrashort-pulse laser fabrication of nanoporous NiO on metal foils | η_10_ - 430 mV  Tafel - 127 mV/dec⁻¹ in 1 M KOH | ^[3]^ |
| NiOx / Ni(OH)2 nanoparticle catalyst layer | Nanoparticle catalyst layer on pretreated working electrode; conventional powder-type catalyst film | η_10_ - 330 mV for NiO_x_, - 300 mV for Ni(OH)_2_ | ^[8]^ |
| Anodized Ni foam in unpurified KOH | Binder-free electro-anodization of Ni foam (higher surface area); Fe taken up in situ from electrolyte | >500 mA cm⁻² at η < 350 mV overpotential (unpurified KOH) | ^[9]^ |
| Fe-doped β-Ni(OH)_2_ nanosheets on Ni foam | Hydrothermal / direct-growth binder-free nanosheets on Ni foam with intentional Fe doping | η_10_ - 219 mV | ^[9]^ |


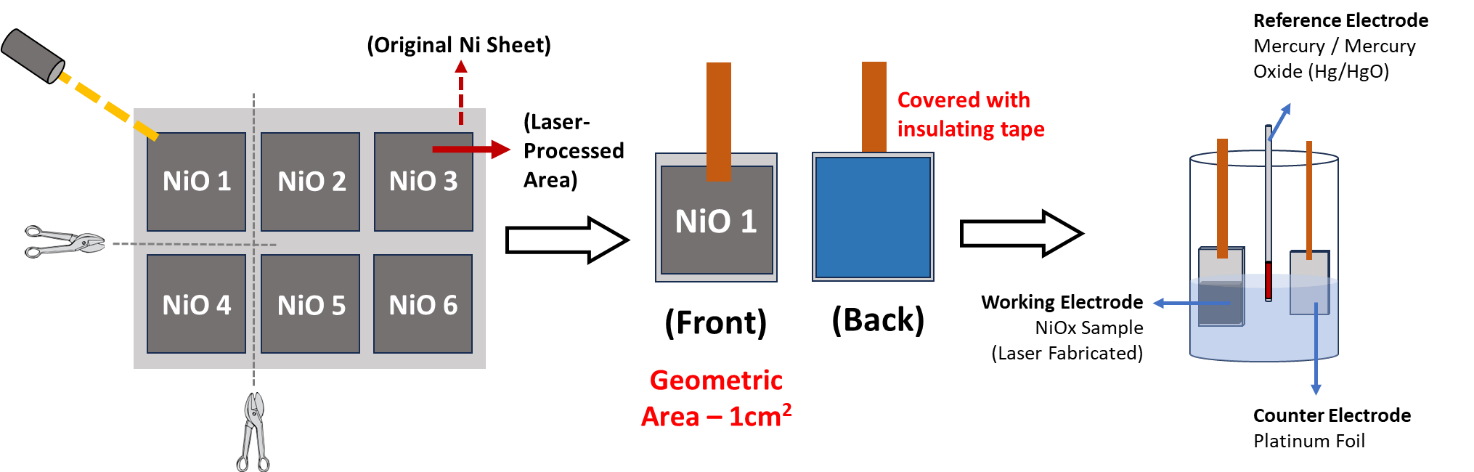


Figure S1 - Schematic showing preparation and ordering of laser-fabricated (ULPING) NiOx sheets for electrochemical tests


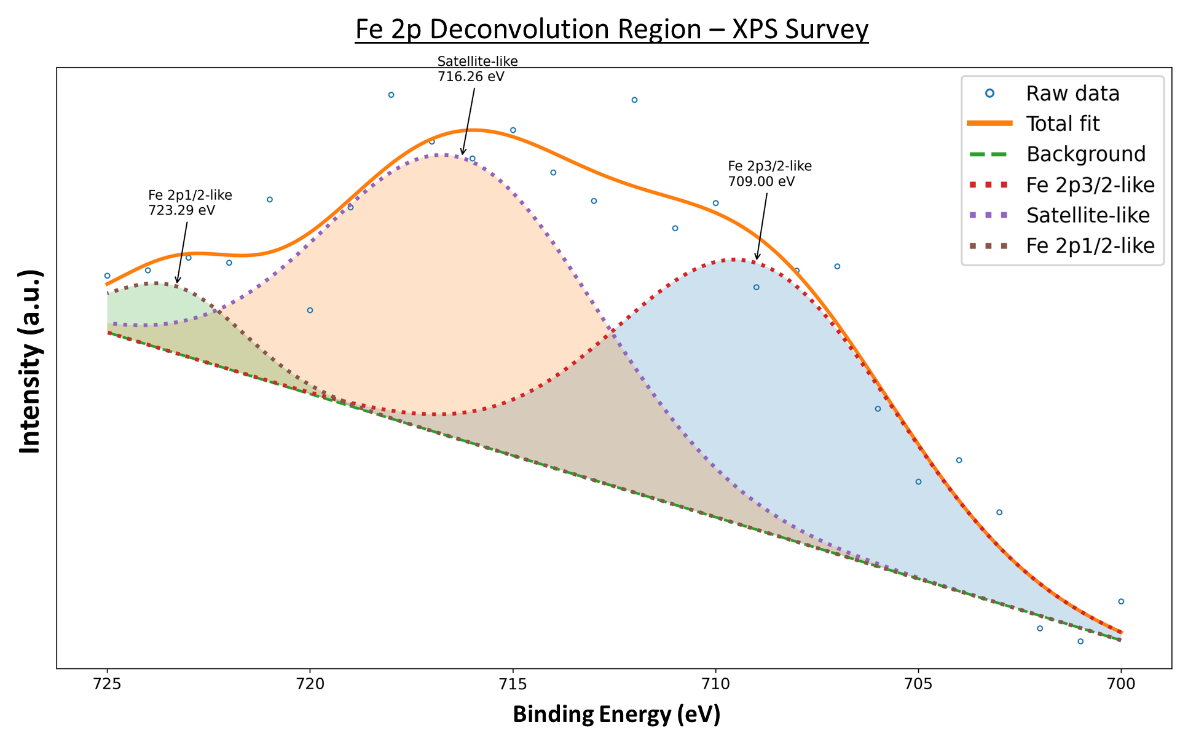


Figure S2- Fe 2p XPS spectra for NiOx (Lower PD) sample after 25 CV cycles. Some kind of peak assignment is made here, after the said deconvolution, however such an ex-situ technique may be unreliable, given that Ni LMM also interferes in this region. This represents the samples to may likely have Fe(II)-like and/or mixed Fe(II)/Fe(III). See the Supporting Note below.

**Supporting Note 1:** Ex-situ XPS and EDX for quantification of surface incorporated Fe on NiOx

Ex-situ conventional XPS may be used for a semi-qualitative analysis of Fe-incorporation in NiOx/NiOOH, but it has clear limitations for trace, dynamic Fe from KOH electrolyte. Fe amount can be extremely small and surface-localized and most laboratory XPS has practical detection limits around ~0.1 at.%. The major issue is also about spectral overlap. For a standard Al Kα source, the Fe 2p region overlaps the Ni LMM Auger feature, making trace-Fe fitting on Ni-based oxides unreliable. Some specific studies explicitly avoided this by using Mg Kα as radiation source or alternative regions such as Fe 3p instead of relying only on Fe 2p. ^[10]^

SEM-EDX is even less suitable for the initial incorporation stage because it samples a much larger interaction volume and has poorer sensitivity for trace and near-surface Fe. SEM-EDX could be used for pre-constructed thicker Fe layers or NiFe alloys. For dynamic tracking of Fe incorporated in such conditions, it is highly favorable and reliable to make use of operando or in-situ techniques such as operando Raman or XANES for accurate establishment of mechanistic trends.

However, after repeated CV cycling and longer galvanostatic (or potentiostatic) hold, Fe can become more accumulated or deeply distributed within the electrochemically accessible oxyhydroxide phase (and even get incorporated in the sub-layers and the bulk) making ex-situ XPS more likely to detect it.^[11]^ Some studies do report Fe in post-conditioned (post-cycled) NiOx from XPS, and we make a similar attempt in this work.

**Supporting Note 2:** Calculating Ablation Depth, Nanostructure Growth Height and Nano Gain Area

The workflow treated pulse duration as the controlling process variable and linked it to local energy delivery, material removal, and morphology build-up. Shorter pulse durations produce shallower crater removal but larger vertical nanostructure growth, whereas longer pulse durations favor deeper ablation and reduced outward nano-growth. To capture this behavior in a compact engineering model, the calculations were organized in four steps: (i) estimate absorbed pulse energy and effective surface heating, (ii) determine ablation depth from a threshold-based relation, (iii) calculate nanostructure height from the fraction of removed material that is redistributed around the crater, and (iv) convert height and feature size into a nano area gain or roughness factor. A semi-infinite 1D thermal response was assumed near the irradiated surface, with uniform effective energy absorption inside the illuminated zone. Material properties were treated as effective constants for each calculation step. The model was not intended to resolve plasma formation, dynamic reflectivity, recoil pressure, or oxidation kinetics explicitly. Nanostructures were represented by an average feature geometry, so the reported growth height and nano area gain correspond to morphology-level engineering descriptors rather than atomistic quantities.

The absorbed fluence was first written in terms of pulse energy and beam radius. Then, a logarithmic ablation law was used to obtain the crater depth per pulse. In the code, the threshold fluence was allowed to vary with pulse duration so that the model could reproduce the experimentally expected dependence of material removal on pulse width.

F_abs = (1 − R) E_p / (π w_0²)

δ(τ_p) = δ_eff ln(F_abs / F_th(τ_p)) for F_abs > F_th

F_th(τ_p) = F_th,ref (τ_p / τ_ref)^(1/2)^

Here, F_abs is the absorbed fluence, R is reflectivity, E_p is pulse energy, w_0 is beam radius, δ is ablation depth, δ_eff is an effective absorption/removal length scale, and F_th is the pulse-duration-dependent threshold fluence.

The nano-growth height was calculated from a local mass/volume balance. Only a fraction of the removed material contributes to useful vertical growth near the crater rim or textured zone, while the remainder is lost through melt flow, vapor ejection, or non-local redistribution. This was represented by a redeposition efficiency term.

h(τ_p) = η_dep(τ_p) δ(τ_p) / φ

η_dep(τ_p) = η_0 (τ_ref / τ_p)^m^

In these expressions, h is the average nanostructure growth height, η_dep is the redeposition efficiency, φ is a packing or porosity correction factor, η_0 is a reference redeposition constant, and m is an empirical exponent describing how rapidly growth efficiency decreases as pulse duration increases.

Once the average height was obtained, the real electrochemically accessible surface area was estimated relative to the projected planar area. For cylindrical or column-like features, the area gain can be written as:

G = A_real / A_proj

G ≈ 1 + n (2π r h)

where G is the nano area gain (roughness factor), n is the feature number density, r is the average nanostructure radius, and h is the previously computed growth height. The first term represents the original planar area, while the second term captures the additional side-wall area generated by the nanostructures. Because h depends strongly on pulse duration, G also becomes pulse-duration dependent. Thus, lower pulse duration gives a larger area gain even when the total ablated depth is smaller.

*References*

[1] K. Khosravinia, A. Kiani, *ACS Omega* **2023**, *8*, 17220.

[2] S. S. Koshy, J. Rath, A. Kiani, *Int. J. Electrochem. Sci.* **2025**, *20*, 101004.

[3] S. S. Koshy, J. Rath, A. Kiani, *Chemical Engineering Journal Advances* **2025**, *24*, 100870.

[4] S. S. Koshy, J. Rath, A. Kiani, *J. Mater. Sci.* **2026**, *61*, 1716.

[5] M. Khot, A. Kiani, *J. Energy Storage* **2022**, *55*, 105779.

[6] M. Khot, A. Kiani, *Lasers in Manufacturing and Materials Processing* **2023**, *10*, 548.

[7] A. Rajurkar, S. Chinchanikar, *International Journal on Interactive Design and Manufacturing (IJIDeM)* **2024**, *18*, 7021.

[8] L.-A. Stern, X. Hu, *Faraday Discuss.* **2014**, *176*, 363.

[9] Y. J. Son, K. Kawashima, B. R. Wygant, C. H. Lam, J. N. Burrow, H. Celio, A. Dolocan, J. G. Ekerdt, C. B. Mullins, *ACS Nano* **2021**, *15*, 3468.

[10] R. A. Marquez, E. Kalokowski, M. Espinosa, J. T. Bender, Y. J. Son, K. Kawashima, C. E. Chukwuneke, L. A. Smith, H. Celio, A. Dolocan, X. Zhan, N. Miller, D. J. Milliron, J. Resasco, C. B. Mullins, *Energy Environ. Sci.* **2024**, *17*, 2028.

[11] F. Bao, E. Kemppainen, I. Dorbandt, F. Xi, R. Bors, N. Maticiuc, R. Wenisch, R. Bagacki, C. Schary, U. Michalczik, P. Bogdanoff, I. Lauermann, R. van de Krol, R. Schlatmann, S. Calnan, *ACS Catal.* **2021**, *11*, 10537.
